# An Evaluation of Co-Occurring Conditions in Newly Diagnosed Adults With Attention Deficit Hyperactivity Disorder (ADHD) in the Brighton and Hove Neurodevelopmental Clinic Between June 2022 and June 2024

**DOI:** 10.1192/bjo.2026.11801

**Published:** 2026-06-30

**Authors:** James Newman, Jessica May, Jessica Eccles, Hussien Elkholy

**Affiliations:** 1 SPFT, Brighton, United Kingdom; 2 Department of Clinical Neuroscience, Brighton and Sussex Medical School, University of Brighton and University of Sussex, Brighton, Brighton, United Kingdom

## Abstract

**Aims:**

ADHD is a neurodevelopmental condition which affects approximately 3-4% of UK adults. ADHD has been linked to a range of co-occurring conditions such as Autism, Substance use disorders and Anxiety.

The aim of this study was to examine the frequency of co-occurring psychiatric conditions among a cohort of adult patients diagnosed with ADHD at the Brighton and Hove Neurodevelopmental Clinic between June 2022 and June 2024.

**Methods:**

Notes of 200 patients, who had received a diagnosis of ADHD by the Neurodevelopmental service (NDS) in Sussex Partnership Foundation Trust (SPFT) between June 2022 and June 2024, were reviewed using the Electronic Health Record system used by SPFT, and by using the Plexus software to review GP records.

We reviewed; ADHD referral, assessment, the first 3 NDS reviews post assessment, each patient’s GP record and any previous psychiatric clinic letters that were available to collect data on co-occurring or historic conditions.

Conditions that we recorded included current or historical substance misuse, Autism, Bipolar affective disorder (BPAD), Major Depressive Disorder (MDD), Generalised Anxiety Disorder (GAD), Post traumatic Stress Disorder (PTSD), Personality disorder (any kind and either an active or historic diagnosis). We also collected data on any history of psychosis.

This study was registered with and approved by SPFT Quality improvement support team (QIST).

**Results:**

Of the 200 records reviewed 173 had ADHD referrals, assessments and diagnoses. Of those records the mean age at diagnosis was 31.9 years, with a median age of 30.

The patients records included showed current or historical evidence of the above conditions in the following proportions; Substance misuse 29%, Autism 38% with a further 5.7% awaiting assessment, BPAD 7%, MDD 17.9%, GAD 18.5%, PTSD 19%, Personality disorder 19% and Psychosis 4.6%.

**Conclusion:**

Our findings provided further evidence of the high rate of co-occurring Autism, Substance misuse, anxiety and depression in those who receive a diagnosis of ADHD in adulthood. This highlights complexity and burden of having ADHD diagnosis. It also indicates the need of revising the service model to ensure an appropriately trained workforce is able to provide comprehensive assessment and diagnosis as this is likely to provide patients with a better experience of care and outcomes.

